# Recent Developments in Tandem White Organic Light-Emitting Diodes

**DOI:** 10.3390/molecules24010151

**Published:** 2019-01-02

**Authors:** Peng Xiao, Junhua Huang, Yicong Yu, Baiquan Liu

**Affiliations:** 1 School of Physics and Optoelectronic Engineering, Foshan University, Foshan 528000, China; xiaopeng@fosu.edu.cn (P.X.); jamha1212@163.com (J.H.); 2 LUMINOUS! Centre of Excellent for Semiconductor Lighting and Displays, School of Electrical and Electronic Engineering, Nanyang Technological University, Nanyang Avenue, Singapore 639798, Singapore; 3 Institute of Polymer Optoelectronic Materials and Devices, State Key Laboratory of Luminescent Materials and Devices, South China University of Technology, Guangzhou 510640, China

**Keywords:** tandem, organic light-emitting diode, white, charge generation layer, doping-free

## Abstract

Tandem white organic light-emitting diodes (WOLEDs) are promising for the lighting and displays field since their current efficiency, external quantum efficiency and lifetime can be strikingly enhanced compared with single-unit devices. In this invited review, we have firstly described fundamental concepts of tandem device architectures and their use in WOLEDs. Then, we have summarized the state-of-the-art strategies to achieve high-performance tandem WOLEDs in recent years. Specifically, we have highlighted the developments in the four types of tandem WOLEDs (i.e., tandem fluorescent WOLEDs, tandem phosphorescent WOLEDs, tandem thermally activated delayed fluorescent WOLEDs, and tandem hybrid WOLEDs). Furthermore, we have introduced doping-free tandem WOLEDs. In the end, we have given an outlook for the future development of tandem WOLEDs.

## 1. Introduction

In recent years, organic light-emitting diodes (OLEDs) have entered the mainstream display market, since they can show comparable performance with the liquid crystal displays [1,2,3,4,5]. Besides, OLEDs are promising for the solid-state lighting, which may be able to compete with GaN-based LEDs [6,7,8,9,10]. This is because OLEDs possess many merits, including high efficiency, low power consumption, broad viewing angle, fast response, thin thickness, solution-processed compatibility as well as flexibility [11,12,13,14,15,16]. With the evolution of emitters and enhancement of device engineering, both phosphorescent and thermally activated delayed fluorescence (TADF) materials-based OLEDs can realize a theoretical unity internal quantum efficiency (IQE) due to the harvest of totally singlet and triplet excitons [17,18,19,20]. For phosphorescent materials, they harness singlets and triplets due to the heavy-atom effect [21,22,23]. In terms of TADF emitters, a small energy gap for triplet excited state (T_1_) and singlet excited state (S_1_) is required, which is beneficial to the reverse intersystem process for the 100% exciton harvesting efficiency [24,25,26].

To further promote the OLED technology for high-quality displays and energy-saving lighting field, white OLEDs (WOLEDs) have vastly attracted both industrial and academic interest [27,28,29,30,31]. In 1994, the first WOLED was developed by Kido and coworkers, showing a maximum power efficiency (PE) of 0.83 lm W^−1^ [32,33]. Over the last twenty-four years, the performance of WOLEDs has been remarkably enhanced. By dint of phosphorescent emitters and advanced outcoupling technique, the PE of WOLEDs could show a PE of 123.4 lm W^−1^ at the illumination-related luminance of 1000 cd m^−2^ with an external quantum efficiency (EQE) of 54.6%, which remains 106.5 lm W^−1^ at an ultrahigh luminance of 5000 cd m^−2^ [34]. Even for flexible phosphorescent WOLEDs, their PE can overtake 100 lm W^−1^ [35]. Currently, the EQE of TADF emitter-based WOLEDs has also been demonstrated to exceed 20% [36,37,38,39]. Besides, the luminance >100,000 cd m^−2^ [40], color rendering index (CRI) >90 [41,42,43,44,45,46], correlated color temperature (CCT) mimicking sunlight (2500–8000 K) [47,48,49], and extremely stable color without variation of Commission International de L’Eclairage (CIE) chromaticity coordinates for WOLEDs have also been investigated [50,51,52]. Hence, these superior properties have demonstrated the great potential of the WOLED technology in the application of displays and lighting.

For the real commercialization, the lifetime is an essential parameter for WOLEDs (i.e., ≥10,000 h at ≥1000 cd m^−2^), aside from the general target for the PE of 40–70 lm W^−1^ [53,54,55,56,57,58]. In the case of fluorescent WOLEDs, their lifetime could overtake 150,000 h at 1000 cd m^−2^ [59]. However, the EQE, current efficiency (CE) and PE are unsatisfactory. To boost the lifetime and efficiency simultaneously, the introduction of tandem device architectures is very helpful [60,61,62,63,64]. This is because the lifetime of tandem OLEDs can be N-fold (N, the number of electroluminescent (EL) units) enhanced, since N-fold luminance can be achieved with the current density similar to that of single-unit OLEDs [65,66,67,68]. As a consequence, the EQE and CE can be also N-fold improved. With the careful manipulation of charge generation layers (CGLs), the PE is possible to be increased [69,70,71]. Therefore, tandem OLEDs have aroused both academic and industry interest. In 2005, Ma et al. reported the first tandem WOLEDs, where the CE and brightness equal basically to the sum of the two EL units [72]. Since then, a large number of endeavors have been taken to tandem WOLEDs [73,74,75,76,77]. As a matter of fact, the current commercial WOLEDs are almost based on tandem architectures. With the development of tandem WOLEDs, the fluorescent, phosphorescent and TADF emitters have been well connected with CGLs to realize desirable performance.

Herein, the fundamental concepts of tandem device architectures and their use in WOLEDs will be firstly described. Then, the state-of-the-art strategies to achieve high-performance tandem WOLEDs in recent years will be summarized. Specifically, the research progresses in the four types of tandem WOLEDs (i.e., tandem fluorescent WOLEDs, tandem phosphorescent WOLEDs, tandem TADF WOLEDs, and tandem hybrid WOLEDs) will be highlighted. Furthermore, doping-free tandem WOLEDs will be introduced. Finally, an outlook for the future development of tandem WOLEDs will be presented.

## 2. Fundamental Concepts of Tandem WOLEDs 

### 2.1. The Device Architecture of Tandem OLEDs

**Figure 1:**
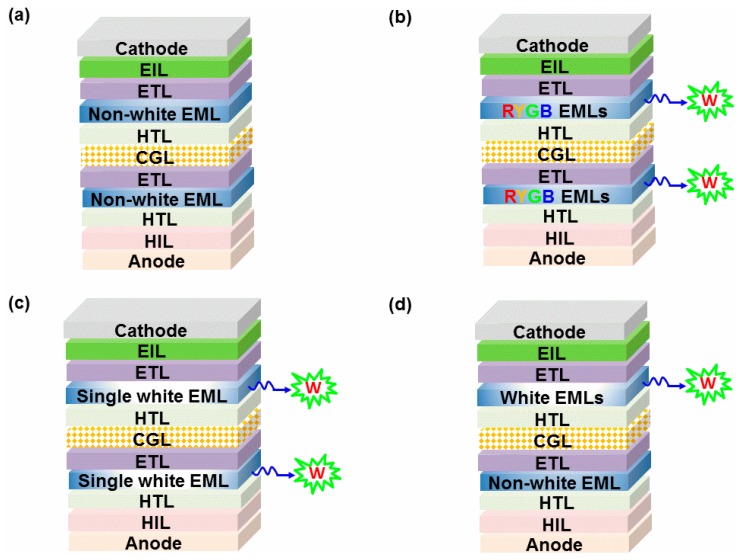
Typical device architectures for tandem white organic light-emitting diodes (WOLEDs). (**a**) Type-I tandem WOLED architecture, in which the white emission is generated via the combination of nonwhite or monochromatic emission from each electroluminescent (EL) unit. (**b**) Type-II tandem WOLED architecture, where RYGB emitting layers (EMLs) mean that more than two EMLs generating white emission in each EL unit. (**c**) Type-III tandem WOLED architecture, where each EML of the EL unit is single white EML. (**d**) Type-IV tandem WOLED architecture, where the non-white EML unit is combined with single white EML or RYGB EMLs. W is white emission, HIL is hole injection layer, HTL is hole transport layer, ETL is electron transport layer, EIL is electron injection layer.

To furnish white emissions, complementary-color emitters, three-color emitters or four-color emitters (e.g., blue/yellow, blue/orange, blue/red, blue/green/red, blue/yellow/red, blue/green/yellow/red) are commonly required [78,79,80,81,82,83,84,85]. For single-unit WOLEDs, the emitting layers (EMLs) can be composed of single or multiple EMLs. In general, single-EML WOLEDs only need one EML comprised of a versatile host doped with different color guests, while multiple-EML WOLEDs require at least two EMLs to produce white emissions [86,87,88,89,90,91]. Accordingly, the EL unit of tandem WOLEDs could be comprised of non-white or monochromatic EMLs, where various-color EMLs are located in different EL unit to combine for the white emission (type-I tandem WOLED architecture), as shown in Figure 1a. Besides, each unit of tandem of WOLEDs can be made up of multiple-EML white unit (type-II tandem WOLED architecture, Figure 1b) or single-EML white unit (type-III tandem WOLED architecture, Figure 1c). Alternatively, two unit-based tandem WOLEDs can be constructed by a white unit and a non-white unit (type-IV tandem WOLED architecture, Figure 1d). Based on the above facts, highly efficient phosphorescent and TADF emitters are usually adopted in tandem WOLEDs, which is expected to harvest both singlet and triplet excitons for the high performance [92,93,94,95,96,97]. After the optimization of EMLs, CGLs are selected to interconnect each EL unit. As a result, tandem WOLEDs will be formed. It is deserved to note that most of tandem WOLEDs are focused on two or three EL units, although more than four units can be connected with CGLs.

### 2.2. The Role of Charge Generation Layer

To guarantee the high performance, the CGL plays a vital role in tandem WOLEDs, apart from the efficient each EL unit [98,99,100]. In 2003, Kido et al. reported the first tandem OLED by using n-doped organic/transparent conductive layer (i.e., Cs:2,9-dimethyl-4,7-diphenyl-1,10-phenanthroline (BCP)/indium tin oxide (ITO)) and n-doped organic/insulating materials (Cs:BCP/V_2_O_5_ or Cs:BCP/4F-TCNQ) as the CGLs [98]. Since then, numerous effective CGLs have been demonstrated. Briefly, a CGL acts as internal anode as well as cathode to generate charges and separate the opposite charges injecting to nearby EL unit [101]. Hence, tandem OLEDs can possibly convert one injected electron to multiple photons, obtaining higher luminance and CE at low current density. Such effect is beneficial to prolong the lifetime due to the avoidance of current leakage and breakdown caused by electric field [102,103,104,105].

For the formation of a CGL, a n-p semiconductor heterojunction is typically needed for the charge generation, which is located at the interface of n-type and p-type layers [106,107,108,109,110]. Besides, some demands are required for an efficient CGL, including excellent charge generation, small barrier for charge separation and injection into the nearby EL unit, outstanding transparency for visible emissions (e.g., ≥75%), good conductivity and long working stability [111,112,113]. So far, a large number of CGLs have been put forward. Apart from the above mentioned n-doped organic material/metal oxide CGL [98], n-doped organic material/p-doped organic material CGL (e.g., Li:tris(8-quinolinolato) aluminum(III) (Alq_3_)/FeCl_3_:4,4′-*N*,*N*’-bis[*N*-(1-naphthyl)-*N*-phenylamino]biphenyl (NPB) [66], 4,7-diphenyl-1,10-phenanthroline (BPhen):Rb_2_CO_3_/NPB:ReO_3_ [114], Bphen:Rb_2_CO_3_/ReO_3_/NPB:ReO_3_ [114]) and organic heterojunction CGL (e.g., copper hexadecafluorophthalocyanine (F16CuPc)/copper phthalocyanine (CuPc) [115], fullerene (C60)/pentacene [116], zinc phthalocyanine (ZnPc):C60 [117]) are the most popular types.

In the physical processes of CGLs, charge generation and charge separation are the two main features. Hence, upon charges generating at the n-p junction, electrons and holes should be rapidly injected into nearby EL unit. To guarantee such charge separation, the CGL materials with high charge mobility or conductivity are ideal [118,119,120,121,122]. Besides, the energy barrier between CGLs and ETL or HTL in each EL unit should be minimized, which cannot only increase the charge injection into the EL unit but also reduce the charge accumulation [123,124,125,126]. To enhance the PE, the CGL is required to possess outstanding charge generation as well as charge separation. Particularly, negligible voltage drop across the CGL in the process of charge generation is required, otherwise the PE cannot be largely enhanced. This is because the voltage of tandem OLEDs usually increases N-fold compared with single-unit devices, leading to the fact that the PE of tandem OLEDs is similar to that of single-unit OLEDs.

## 3. Stratagies for Tandem WOLEDs

### 3.1. Basic Aspects of Tandem WOLEDs 

Based on the employed emitters, tandem WOLEDs can be classified into four types, i.e., tandem fluorescent WOLEDs, tandem phosphorescent WOLEDs, tandem TADF WOLEDs, and tandem hybrid WOLEDs. Thus, the selection of efficient emitters is essential to the high performance [127,128,129,130,131,132]. Unlike single-unit WOLEDs, the adoption of effective CGLs is another essential for high-performance tandem WOLEDs according to the above mentioned concepts. Therefore, the core features of strategies for tandem WOLEDs are the simultaneous management of efficient emitters and effective CGLs. To ensure the efficient emission can be produced in each EL unit of tandem WOLEDs, the careful manipulation of charges and excitons distribution is necessary [133,134,135,136,137,138]. Specifically, the issues of charge injection from the electrodes, charge transport and charge balance in EMLs, exciton generation, exciton harvesting, exciton recombination, exciton diffusion and exciton quenching should be well manipulated [139,140,141,142]. On the other hand, the CGL should not only be effective to generate and separate charges but also be able to optimize the optical effect to reduce the light loss. In the following sections, the design strategy, device architecture, emission mechanism as well as the effect of CGL in tandem WOLEDs have been highlighted, including tandem fluorescent, phosphorescent, TADF and hybrid WOLEDs. Additionally, the application of doping-free technique in tandem WOLEDs has also been described.

### 3.2. Tandem Fluorescent WOLEDs

**Figure 2:**
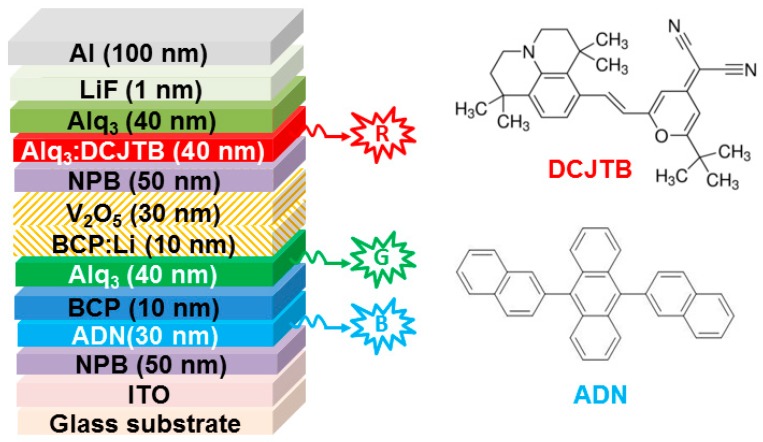
**Right**: the device structure of tandem WOLEDs. R, G and B represent red, green and blue emission, respectively. **Left**: the molecular structures of 4-(dicyanomethylene)-2-t-butyle-6-(1,1,7,7-tetramethyljulolidyl-9-enyl) 4H-pyran (DCJTB) and 9,10-bis-(β-naphthyl)-anthrene (AND) [72].

The first tandem WOLED was based on fluorescent emitters, in which 4-(dicyanomethylene)-2-t-butyle-6-(1,1,7,7-tetramethyljulolidyl-9-enyl) 4H-pyran (DCJTB), Alq_3_ and 9,10-bis-(β-naphthyl)-anthrene (ADN) were used as the red (600 nm), green (505 nm) and blue (435 nm) fluorescent emitter, respectively [72]. The device structure was ITO/NPB (50 nm)/ADN (30 nm)/BCP (10 nm)/Alq_3_ (40 nm)/BCP:Li (10 nm)/V_2_O_5_ (30 nm)/NPB (50 nm)/Alq_3_:DCJTB (40 nm)/Alq_3_ (40 nm)/LiF (1 nm)/Al (200 nm), which can be classified into type-I tandem WOLED architecture, as shown in Figure 2. As a result, the tandem WOLED showed a stable white light with CIE coordinates from (0.35, 0.32) at 18 V to (0.36, 0.36) at 50 V. The maximum luminance of 10,200 cd m^−2^ and CE was 10.7 cd A^−1^, which were equal basically to the sum of the two EL units. For the origin of strikingly enhanced performance, the CGL of BCP:Li (10 nm)/V_2_O_5_ (30 nm) played a key role. In brief, electrons and holes were generated within the CGL and then reached nearby Alq_3_ and NPB, respectively. On one hand, these electrons could arrive at the blue EML ADN to recombine with holes injected from anode, generating blue emission. Since BCP is a hole-blocking material, the holes distribution could be manipulated by adjusting the thickness of BCP layer, assuring blue and green emissions. On the other hand, holes generated from the CGL could reach the red EML Alq_3_:DCJTB and recombine with electrons injected from cathode, producing the red emission. Therefore, white emission has been achieved.

After Ma’s pioneering work [72], many efforts have been made on tandem fluorescent WOLEDs [143]. For example, Ho et al. used n-doped organic material/p-doped organic material CGL (i.e., Bphen:2% cesium carbonate (Cs_2_CO_3_)/NPB:50% *v*/*v* tungsten oxide (WO_3_)) to interconnect blue fluorescent EL unit and red fluorescent EL unit (type-I tandem WOLED architecture) [144]. By virtue of the p-i-n technology, their device showed a CE of 23.9 cd A^−1^ and PE of 7.8 lm W^−1^, which are one of the best for tandem fluorescent WOLEDs. However, despite the luminance and efficiency of tandem fluorescent WOLEDs can be scale linearly with the number of EL units, triplet excitons still decay nonradiatively, leading to the fact that the performance of this kind of devices is not satisfactory enough [145,146,147,148,149]. Therefore, more works are focused on phosphorescent or TADF emitters-based tandem WOLEDs.

### 3.3. Tandem Phosphorescent WOLEDs

In the case of phosphorescent emitters, triplet excitons can be harvested via the triplet-triplet energy transfer, while singlet excitons are harnessed via the singlet-triplet intersystem crossing process due to the heavy-atom effect, leading to a maximum IQE of 100% [150]. As an excellent result, single-unit phosphorescent WOLED with fluorescent tube efficiency has been demonstrated [151]. With the improvement of material design and device engineering, it is not very surprising to see that the EQE of single-unit phosphorescent WOLED can be >20% currently [152,153,154]. By extending the application of phosphorescent emitters into tandem WOLEDs, high-performance devices can be also developed with the management of effective CGLs.

**Figure 3:**
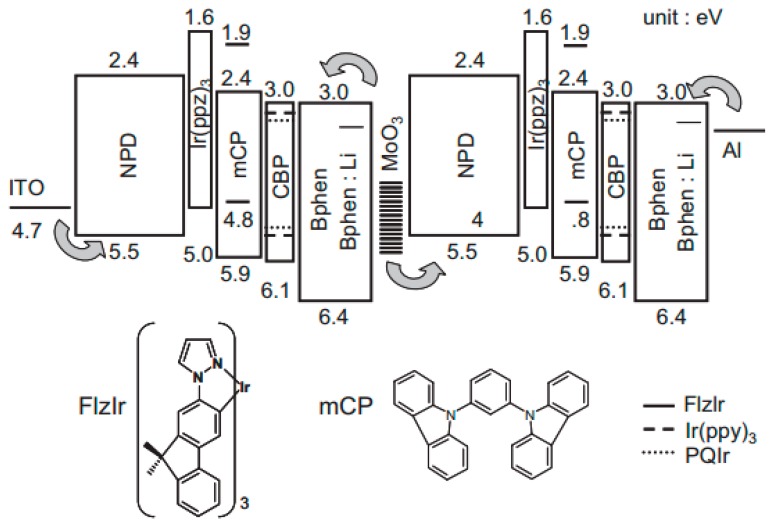
**Top:** Proposed energy-level diagram of tandem WOLEDs. Numbers indicate the HOMO and LUMO energies relative to vacuum. The arrows indicate the carrier injection from electrodes and the MoO_3_ CGL. **Bottom:** The molecular structures of Ir(flz)_3_ and mCP. Reproduced from reference [155].

In 2006, Kanno et al. reported the first tandem phosphorescent WOLED [155]. By using a n-doped organic material/metal oxide CGL (i.e., Bphen:Li/MoO_3_) to interconnect the double-EML white EL unit (i.e., 10 wt. fac-tris(1-(9,9-dimethyl-2-fluorenyl)pyrazolyl*N,C2*′)iridium(III) (Ir(flz)_3_):*N,N*′-dicarbazolyl-3,5-benzene (mCP, 20 nm)/10 wt.% iridium(III) bis(2-phenylquinolyl-*N,C2*′)acetylacetonate (PQIr):3 wt.% fac-tris(2-phenylpyridinato-*N,C2*′)iridium(III) (Ir(ppy)_3_):4,4′-*N*,*N*′-dicarbazole-biphenyl (CBP, 5 nm)), tandem WOLEDs with 2 and 3 EL units were developed, which can be classified into type-II tandem WOLED architecture, as shown in Figure 3. As a result, the tandem WOLED with 3 EL units reached a peak forward-viewing EQE of 34.9% and total EQE of 51.0% at 500 cd m^−2^. Briefly, there were four factors for the high performance. (i) The Li doping of Bphen in the CGL allowed for efficient electron injection, otherwise inefficient charge generation would occur. (ii) Charges and excitons were confined within the EMLs of each subpixel in the stack, by using materials with wide energy gaps i.e., fac-tris(1-phenypyrazolyl-*N*,*C2*′)iridium(III) (Ir(ppz)_3_) and Bphen as barriers to exciton and charge diffusion across the EML/HTL and EML/ETL interfaces, respectively. (iii) All exciton formation occurred within each EML by direct excitation of the triplet, either by injection from the lowest unoccupied molecular orbital (LUMO) CBP or by electron transfer from the mCP LUMO. (iv) Optical interference and weak microcavity effects were controlled by varying the thickness of HTL 4,4′-bis[*N*-(1-naphthyl)-*N*-phenyl-amino]biphenyl (NPD) with high hole mobility, since its thickness variation (<100 nm) would not significantly affect the voltage nor charge balance and hence the position of the recombination zone. Therefore, the charge confining structure and the effective CGL ensured a uniform white color balance for each subpixel, along with high efficiency for charge injection.

**Figure 4:**
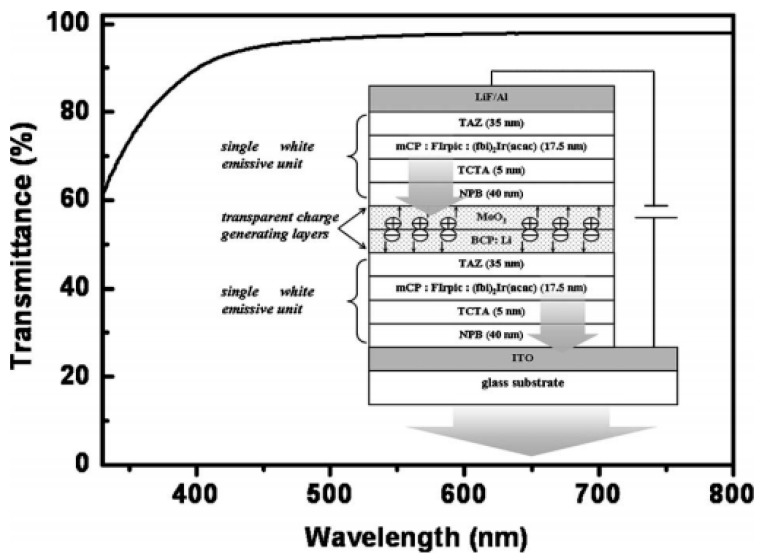
Transmittance spectrum of Li:BCP (10 nm)/MoO_3_ (7 nm) thin film. **Inset**: schematic device structure of the tandem WOLED. Reproduced from reference [162].

In general, the architecture of type-III tandem WOLEDs is simpler than that of type-II tandem WOLEDs due to the reduced number of EMLs. However, it is usually more challenging to achieve high efficiency for single-EML WOLEDs compared with multiple-EML WOLEDs [156,157,158,159,160]. To loosen this bottleneck, Wang et al. have first realized highly efficient single-unit WOLED with the EML of mCP:iridium(III)[bis(4,6-difluorophenyl)-pyridinato-*N*,*C20*]picolinate(FIrpic):bis(2-(9,9-diethyl-9*H*-fluoren-2-yl)-1-phenyl-1*H*-benzoimidazol*N*,*C3*)iridium(acetylacetonate) ((fbi)_2_Ir(acac)), by harvesting all excitons via two parallel channels:host-guest energy transfer for FIrpic and direct exciton formation following charge trapping for (fbi)_2_Ir(acac) [161]. Then, they have used such single-EML architecture as the EL unit of a tandem WOLED via the interconnection of a n-doped organic material/metal oxide CGL (i.e., Li-doped BCP/MoO_3_), which can be classified into type-III tandem WOLED architecture, as shown in Figure 4 [162]. As a result, the tandem WOLED exhibited a maximum forward viewing CE of 110.9 cd A^−1^, EQE of 43.3% and PE of 45.2 lm W^−1^, which were the best for tandem WOLEDs with two EL units at that time. Thus, the high performance was attributed to the combination of effective single units and CGL.

**Figure 5:**
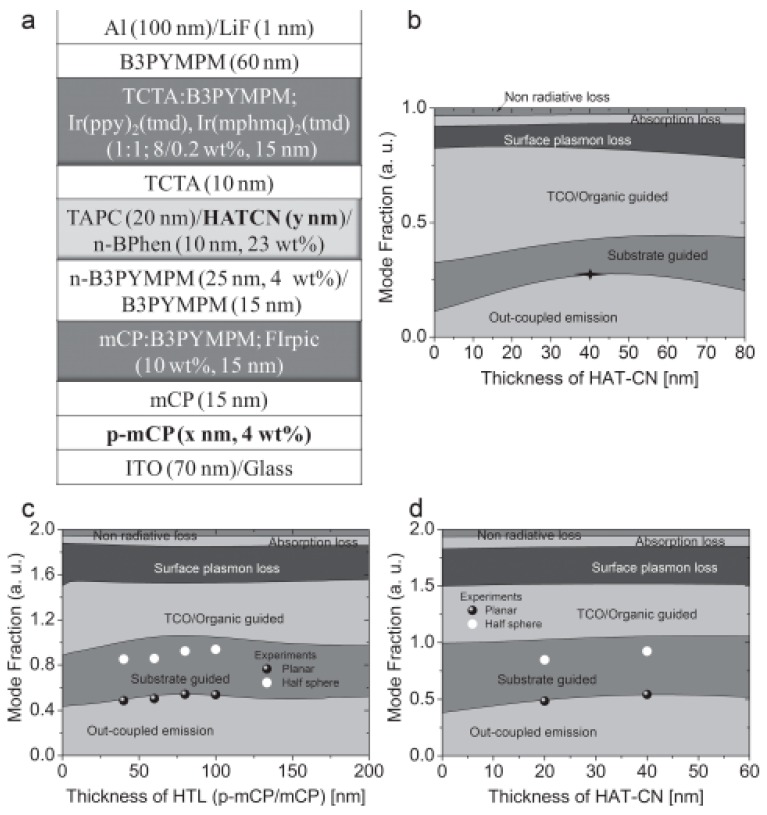
(**a**) Structure of tandem WOLEDs. (**b**) Mode analysis of blue light as a function of HATCN thickness with a total HTL thickness of 80 nm. 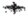 indicates the thickness of HATCN when an out-coupled mode is maximized. (**c**) Mode analysis of white light as a function of the total HTL thickness at an HATCN thickness of 40 nm. (**d**) Mode analysis of white light as a function of HATCN thickness with a total HTL thickness of 80 nm. Reproduced from reference [163].

After the selection of CGLs, the optimization of efficient phosphorescent emitters is key to the performance. Towards this end, Lee et al. used an exciplex-forming co-host and red- and green-phosphorescent dyes with horizontally oriented transition dipoles to optimize an orange OLED with a maximum EQE of 32% [163]. Next, by connecting such efficient orange EL unit with a blue phosphorescent EL unit via a CGL of Rb_2_CO_3_-doped BPhen/1,4,5,8,9,11-hexaazatriphenylene hexacarbonitrile (HATCN)/4,7-diphenyl-1,10-phenanthroline (TAPC), tandem WOLEDs showed a maximum EQE of 54.3% without outcoupling technology or EQE of 90.6% at 1000 cd m^−2^ by attaching an index-matched glass half sphere onto the glass substrate. The device structure was ITO (70 nm)/4% ReO_3_-doped mCP (x nm)/mCP (15 nm)/mCP:bis-4,6-(3,5-di-3-pyridylphenyl)-2-methylpyrimidine (B3PYMPM):FIrpic (15 nm, 0.7:0.3:0.057 molar ratio)/B3PYMPM (15 nm)/4 wt% Rb_2_CO_3_-doped B3PYMPM (25 nm)/23 wt% Rb_2_CO_3_-doped BPhen (10 nm)/HATCN (y nm)/TAPC (20 nm)/4,4′,4″-tri(*N*-carbazolyl)triphen-ylamine (TCTA, 10 nm)/TCTA:B3PYMPM:iridium(III) bis(2-phenylquinoline) tetramethylheptadionate (Ir(ppy)_2_(tmd)):Iridium(III) bis(4-methyl-2-(3,5-dimethylphenyl)quinolinato-*N*,C2′) tetramethylheptadionate (Ir(mphmq)_2_(tmd)) (15 nm, 0.5:0.5:0.1:0.002 molar ratio)/B3PYMPM (60 nm)/LiF (0.7 nm)/Al (100 nm), which can be classified into type-I tandem WOLED architecture, as shown in Figure 5a. A key feature for the high performance was the introduction of the exciplex host TCTA:B3PYMPM for Ir(ppy)_2_(tmd) and Ir(mphmq)_2_(tmd) having highly oriented triplet transition dipole moments along the horizontal direction and high photoluminescence quantum yields (PLQY) [164,165,166,167,168]. Besides, the tandem device architecture was optimized by an optical simulation to maximize the outcoupling of the emitted light. For example, the location for the blue EL unit, orange EL unit and the total thickness of ITO and organic layers have been optimized via the simulation based on the classical dipole model, as shown in Figure 5b–d.

**Figure 6:**
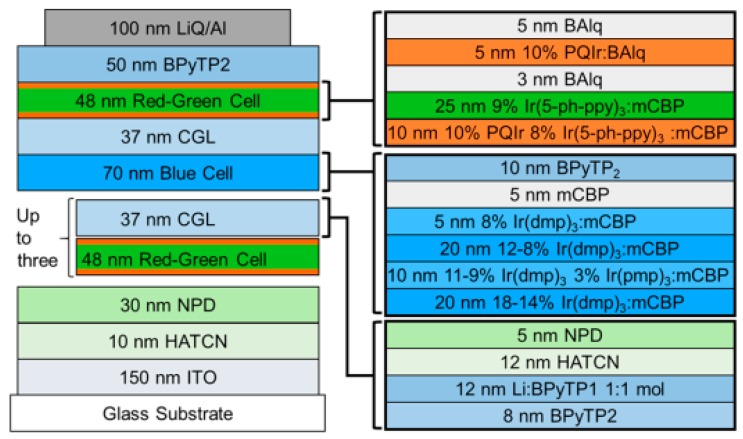
Device structure: (**left**) Tandem WOLEDs D3, D4, and D5 have one, two, and three CGL/red-green element pairs below the blue element, respectively. (**Right**) Layers of the red-green element, blue element and CGL. Reproduced from reference [174].

It is well-known that blue phosphorescent emitters usually exhibit poor stability [169,170,171,172,173]. Hence, the lifetime of tandem phosphorescent WOLEDs is limited. Recently, a strategy to address this issue has been proposed by Coburn et al. [174]. The device structure was glass substrate/150 nm ITO/10 nm HATCN/30 nm NPD/1-3 red-green emitter-CGL pairs (D3-D5, respectively)/blue element/CGL/red-green element/50 nm BPyTP2/1.5 nm 8-hydroxyquinolinato lithium (LiQ)/100 nm Al, where the red-green element was 10 nm 4,40-bis(3-methylcarbazol-9-yl)-2,20-biphenyl (mCBP):8 vol % Ir(5′-Phppy)_3_:10 vol % PQIr/25 nm mCBP:9 vol % Ir(5-Ph-ppy)_3_/3 nm BAlq/5 nm BAlq:10 vol % PQIr/5 nm BAlq, the blue element was 20 nm mCBP:18 → 14 vol % Ir(dmp)_3_/10 nm mCBP:11 → 19 vol % Ir(dmp)_3_:3 vol % mer-tris(*N*-phenyl, Nmethyl-pyridoimidazol-2-yl)iridium(III) (mer-Ir(pmp)_3_)/20 nm mCBP:12 → 8 vol % Ir(dmp)_3_/5 nm mCBP:8 vol % Ir(dmp)_3_/5 nm mCBP/10 nm BPyTP2, the CGL was 8 nm 2,7-bis(2,20-bipyridine-5-yl)triphenylene (BPyTP2)/12 nm BPyTP2:3 vol % Li/12 nm HATCN/5 nm NPD, D3, D4, and D5 indicated the total number of stacked elements separated by CGLs, as shown in Figure 6. As a result, the optimized tandem WOLED D5 showed a CCT of 2780 K, CRI of 89, a peak PE of 50 lm W^−1^ and a T_70_ lifetime (defined as the time corresponding to 30% decrease in luminance from an initial value of 1000 cd m^−2^) of 80,000 ± 2000 h, with minimal spectral shifts during aging. The key features for the high performance were red emissive blocking layers in the red-green element, graded doping and hot excited state management in the blue element, stable and low voltage CGLs, and effective outcoupling technique. More specifically, (i) stable bis(8-hydroxy-2-methylquinoline)-(4-phenylphenoxy)aluminum (BAlq) was employed as a hole blocking layer for green element and a thin spacer between the green EML and red doped region, which could also reduce the loss of excitons transferred to its low triplet energy; (ii) the exciton confinement at the EML interface with NPD was obtained by doping red phosphor into a thin green EML adjacent to HTL; (iii) placing red EMLs on both sides of green EML reduced the color shift; iv) dopant grading balanced hole and electron transport in blue EML, broadening the exciton recombination zone and reducing bimolecular annihilation rates that lead to molecular dissociation, which could increase the blue element stability; (v) mer-Ir(pmp)_3_ was used to improve the reliability of the blue element by reducing the probability that hot excited states degrade host or emitter molecules; (vi) the CGL was stable and possessed high charge mobility; (vii) an outcoupling improvement of 2.2 ± 0.2 times over substrate emission by outcoupling substrate modes using index matching fluid between the device substrate and photodetector during the EQE measurement.

### 3.4. Tandem TADF WOLEDs

For TADF emitters, triplet excitons could be harnessed as delayed fluorescence through their up-conversion from a lowest triplet state to a lowest singlet state by inducing efficient reverse intersystem crossing [175,176,177,178,179,180]. Typically, the energy gap between T_1_ and S_1_ of ≤0.2 eV is favorable to the thermal up-conversion [181,182,183]. Similar to phosphorescent emitters, 100% IQE can be attained for TADF emitters [184,185,186]. Thus, the excellent characteristics of TADF emitters render that they are promising for WOLEDs. Since the first single-unit TADF WOLED reported in 2014 [187], the EQE of TADF-based WOLEDs has been demonstrated to be as high as 20%, which is comparable to state-of-the-art phosphorescent WOLEDs [188,189,190]. So far, two kinds of TADF emitters have been reported, TADF materials and TADF exciplexes [191,192,193,194,195]. In general, these two kinds of emitters can be used to develop high-performance WOLEDs by carefully manipulating the charges and excitons distribution [196].

**Figure 7:**
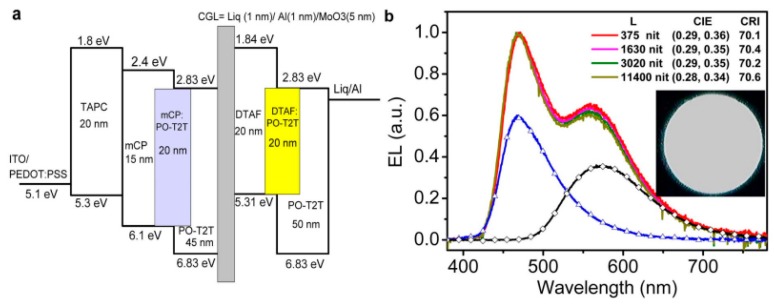
(**a**) Energy levels for the tandem WOLED. (**b**) EL spectra at various luminance, and two decomposed bands were blue and yellow exciplex emissions. Reproduced from reference [197].

By using effective CGLs to interconnect the TADF emitters (TADF materials or TADF exciplexes), tandem TADF WOLEDs can be achieved. Toward this target, Hung and coworkers demonstrated a tandem WOLED, in which blue TADF exciplex EL unit and yellow exciplex EL unit were interconnected by a CGL of 9,9-di[4-(di-p-tolyl)aminophenyl]fluorine (DTAF)/MoO_3_/Al/Liq (holes and electrons were generated from the DTAF/MoO_3_ interface) [197]. The structure was ITO/polyethylene dioxythiophene:polystyrene sulfonate (PEDOT:PSS, 30 nm)/TAPC (20 nm)/mCP (15 nm)/mCP:(1,3,5-triazine-2,4,6-triyl)tris(benzene-3,1-diyl))tris(diphenylphosphine oxide) (PO-T2T) (1:1, 20 nm)/PO-T2T (45 nm)/Liq (1 nm)/Al (1 nm)/MoO_3_ (5 nm)/DTAF (20 nm)/DTAF:PO-T2T (1:1, 20 nm)/PO-T2T (50 nm)/Liq (0.5 nm)/Al (100 nm), as shown in Figure 7. As a result, am EQE of 11.6% was realized. For such high-efficiency exciplex based tandem WOLED, the efficient blue TADF exciplex is important. To accomplish this goal, Hung et al. synthesized PO-T2T having a low HOMO, low LUMO, high T_1_ of 2.99 eV and electron mobility of >10^−3^ cm^2^ V^−1^ s^−1^ as the acceptor. Combined with the mCP donor, the blue TADF exciplex emission could show an EQE of 8.0%, which ensured the high performance of tandem WOLED [188]. In Hung′s device [197], the blue TADF exciplex has been used. To extend this strategy, Zhao et al. used a CGL of 2,4,6-tris(3-(1*H*-pyrazol-1-yl)phenyl)-1,3,5-triazine (3P-T2T):(Cs_2_CO_3_)/Al/MoO_3_ to interconnect both blue TADF exciplex EL unit (TCTA:Bphen) and orange TADF exciplex unit (TAPC:2,4,6-tris(3-(1H-pyrazol-1-yl)phenyl)-1,3,5-triazine), achieving a tandem TADF WOLED with an EQE of 9.17% [198].

### 3.5. Tandem Hybrid WOLEDs

With the combination of blue fluorescent/TADF emitters and green-red/yellow/orange phosphorescent emitters, hybrid WOLEDs can be achieved [199,200,201,202,203,204,205]. The first hybrid WOLED-based on blue fluorescent emitter was reported in 2003 [199]. Then, Sun et al. demonstrated the first efficient hybrid WOLED with a total EQE of 18.7% and a total PE of 37.6 lm W^−1^ [200]. In 2014, Zhang et al. realized the first hybrid WOLED based on blue TADF emitter, achieving a maximum forward-viewing EQE of 22.5% and a peak PE of 47.6 lm W^−1^ [55]. Due to the stable blue fluorescent emitters, most of available products in the WOLED market are adopted the hybrid WOLED technology. Particularly, tandem hybrid WOLEDs are promising for the practical applications.

**Figure 8:**
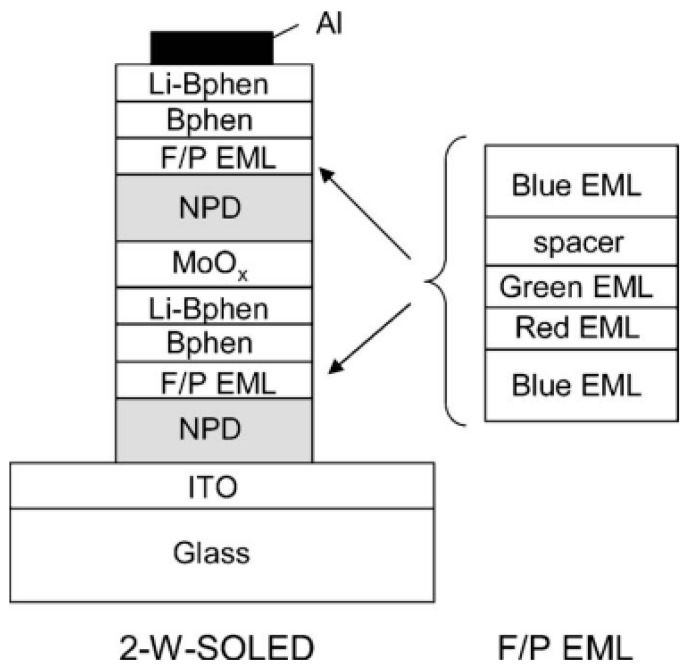
Structure of organic layers of a two-element WOLED. Reproduced from reference [206].

To realize tandem hybrid WOLEDs, Kanno et al. used a CGL of Bphen:Li/MoO_3_ to interconnect two hybrid white EL units [206]. Although this CGL was the same as that of their tandem phosphorescent WOLEDs [155], the tandem hybrid WOLED with three EL units showed a maximum total EQE of 57% at a luminance of 1000 cd m^−2^, representing a 25% increase relative their previous tandem phosphorescent WOLEDs [155]. For the origin of such high performance, an efficient management of singlet and triplet excitons has been accomplished in the EML of each white EL unit, by locating a CBP spacer between blue fluorescent emitting zone CBP:4,4′-bis(9-ethyl-3-carbazovinylene)-1,1′-biphenyl (BCzVBi) and the green and red phosphorescent regions containing CBP:Ir(ppy)_3_ and CBP:PQIr, as shown in Figure 8. Therefore, BCzVBi harvested a majority of singlet excitons, with the remainder of lower energy triplets diffusing through the conductive host CBP to directly excite the green and red phosphors. Such structure allowed high PE via the resonant energy transfer from the conductive host into both the singlet and triplet energy levels. Besides, the NPD thickness in each EL unit was optimized to form the desired white balance in the presence of weak optical interference.

**Figure 9:**
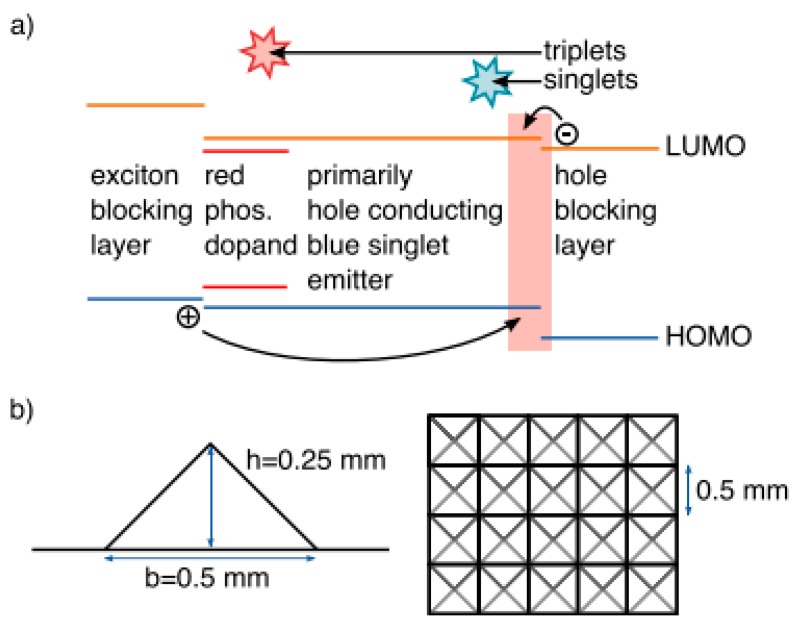
(**a**) Diffusively harvesting triplets. Due to the primarily hole-conducting character of 4P-NPD, excitons were formed close to Bphen. Whereas singlets recombine rapidly after creation, triplets diffuse to Ir(MDQ)_2_acac. (**b**) Patterned surface for enhanced light extraction from the substrate. Reproduced from reference [210].

In Kanno’s tandem hybrid WOLEDs, the T_1_ of BCzVBi is lower than that of phosphorescent emitters. As a result, some triplet excitons are inevitably quenched by the high concentration of blue fluorophor, leading to the fact such device architecture is difficult to achieve 100% EQE [207,208,209]. To ensure the 100% IQE of tandem hybrid WOLEDs, Leo et al. proposed an effective strategy to develop tandem hybrid WOLEDs by using a CGL to interconnect the triplet-harvesting unit and mixed phosphorescent unit, as shown in Figure 9 [210]. In the triplet-harvesting unit, highly efficient fluorescent blue bulk emitter 4P-NPD was used as the blue emitter and host of red phosphorescent emitter Ir(MDQ)_2_acac, since the T_1_ of 4P-NPD (2.3 eV) is higher than that of Ir(MDQ)_2_acac. Due to the p-type property of 4P-NPD, the structure of 65 nm HTL/10 nm Spiro-TAD/5 nm 4P-NPD:5 wt% Ir(MDQ)_2_acac/5 nm 4P-NPD/10 nm BPhen/55 nm BPhen:Cs/100 nm Al rendered that a narrow recombination zone close to the hole blocking layer BPhen. By optimizing the thickness of undoped 4P-NPD layer to be larger than the extension of the recombination zone, Ir(MDQ)_2_acac could only be excited by exciton diffusion owing to the different lifetimes of singlets and triplets (Figure 9). Therefore, the undoped 4P-NPD harvested the singlets, while Ir(MDQ)_2_acac consumed the triplets, producing 100% IQE white emission. In the mixed phosphorescent unit, the structure of 50 nm HTL/10 nm Spiro-TAD/10 nm TCTA:8 wt% Ir(ppy)_3_:1 wt% Ir(dhfpy)_2_acac/10 nm TPBi/50 nm BPhen:Cs/100 nm Al ensured the green and yellow emissions from Ir(ppy)_3_ and Ir(dhfpy)_2_acac, respectively. The two phosphorescent emitters mixed in a common matrixTCTA were without loss of efficiency, which also reduced the voltage due to the reduction from two to one EML. As a consequence, both of the individual units had the ability to reach a 100% IQE. By stacking both units using a CGL consisting of a p/n-junction with a thin metal layer in between, the resultant architecture was 45 nm HTL (HTL1)/10 nm Spiro-TAD/5 nm 4P-NPD:5 wt% Ir(MDQ)_2_acac/5 nm 4P-NPD/10 nm BPhen/90 nm BPhen:Cs(ETL1)/0.5 nm Al/85 nm HTL (HTL2)/10 nm Spiro-TAD/10nm 8 wt% Ir(ppy)_3_:1 wt% Ir(dhfpy)_2_acac/10 nm TPBi/60 nm BPhen:Cs (ETL2)/100 nm Al. Thus, such device can be classified into type-IV tandem WOLED architecture. The PE and EQE were 33 lm W^−1^ and 26%, respectively. Furthermore, by using a quadratic pyramid pattern and a high-refractive-index hemisphere to harvest all the light coupled into the substrate, silver cathode to reduce the absorption, optimized thickness of each transport layer, a PE of 90.5 lm W^−1^ and EQE of 75.8% at 1000 cd m^−2^ were obtained with the help of strongly increased light extraction. By extending this design strategy, Leo et al. have then developed efficient color stable inverted top-emitting tandem hybrid WOLEDs with ultra-thin wetting layer top electrodes [211], ITO-free tandem hybrid WOLEDs with angular color stability [212] and top-emitting tandem hybrid WOLEDs comprising laminated microlens films [213].

**Figure 10:**
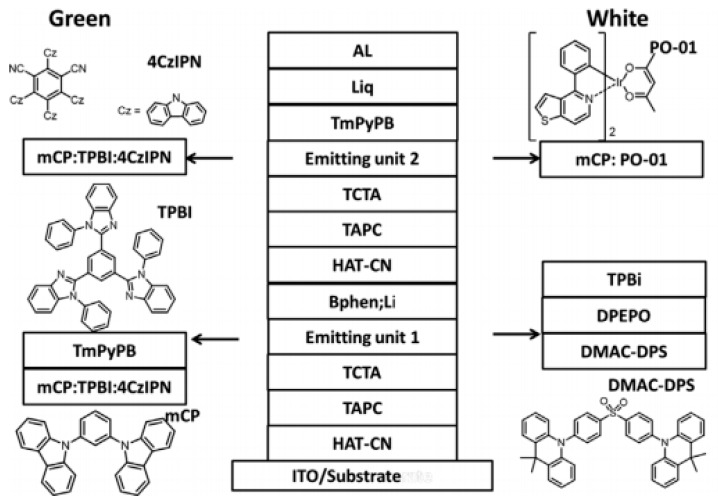
Device configurations of green (G1) and white (W1) OLEDs. Key molecular materials used for green emission (**left**) and those used for white emission (**right**) are shown. Reproduced from reference [216].

Since blue TADF emitters can produce highly efficient blue emission by harvest both singlet and triplet excitons, such kind of emitters have great potential to develop tandem hybrid WOLEDs [214,215]. Recently, Hung et al. reported the first tandem hybrid WOLED by utilizing blue TADF emitter bis[4-(9,9-dimethyl-9,10-dihydroacridine)phenyl]sulfone (DMAC-DPS) and orange phosphorescent emitter:bis(4-phenylthieno-[3,2-c]pyridine) (acetylacetonate)iridium(III) (PO-01), showing the peak CE of 78.5 cd A^−1^ and EQE of 28.5% with the CIE coordinates of (0.33, 0.45) at 1000 cd m^−2^ [216]. The device structure was ITO/HAT-CN (10 nm)/TAPC (40 nm)/TCTA (10 nm)/DMAC-DPS (20 nm)/DPEPO (5 nm)/TPBI (40 nm)/Bphen:Li (1.2 wt.%, 15 nm)/HAT-CN (10 nm)/TAPC (40 nm)/TCTA (10 nm)/mCP:PO-01 (1.4 wt.%, 20 nm)/1,3,5-tri[(3-pyridyl)-phen-3-yl (TmPyPB, 40 nm)/Liq (2 nm)/Al (120 nm), as shown in Figure 10. Prior to the tandem hybrid WOLED, Hung et al. realized an efficient tandem green TADF OLED with the CGL of Bphen:Li/HAT-CN, achieving the EQE of 32.5%. Such results also demonstrated the CGL was effective, which was attributed to the efficient charge generation, excellent optical transparency and good electron transporting properties of Bphen; Li. On the other hand, since both blue TADF emitter and phosphorescent emitter could harvest singlets and triplets in their individual EL unit, high efficiency was attained.

### 3.6. Doing-Free Tandem WOLEDs

Tandem WOLEDs can greatly boost the performance, however, their structures are intrinsically complicated compared with those of single-unit OLEDs. Additionally, the doping technology is required for high-performance tandem WOLEDs (e.g., the p-doping and n-doping charge transport layers, doping different-color EMLs, doping CGLs), which further complicating the structures [217,218,219,220,221]. To simplify the tandem WOLEDs, the doping-free technique may be conducive, since it can simplify the device engineering, shorten the fabrication procedure, avert the utilization of host and lower the cost [222,223,224].

**Figure 11:**
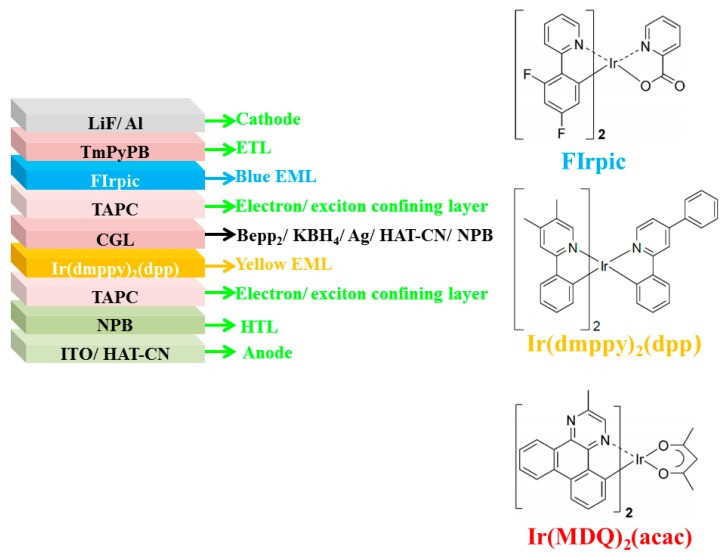
(**Left**): The structures of tandem WOLEDs. (**Right)**: The chemical structure of used emitters. Reproduced from reference [224].

In 2007, Liu et al. demonstrated the doping-free tandem WOLEDs by managing an effective doping-free CGL of Bepp_2_ (25 nm) /KBH_4_ (1 nm)/Ag (0.5 nm)/HAT-CN (130 nm)/NPB (15 nm) to interconnect doping-free EMLs and doping-free charge transport layers [224]. The device architecture was ITO/HAT-CN (100 nm)/NPB (15 nm)/TAPC (5 nm)/bis(2-phenyl-4,5-dimethylpyridinato)[2-(biphenyl-3-yl)pyridinato] iridium(III) (Ir(dmppy)_2_(dpp), 0.6 nm)/CGL/TAPC (5 nm)/FIrpic (0.5 nm)/TmPyPB (50 nm)/LiF (1 nm)/Al (200 nm), where Ir(dmppy)_2_(dpp) and FIrpic were the yellow and blue emitters (device W1), respectively, as shown in Figure 11. Additionally, by using the red emitter Ir(MDQ)_2_(acac) to replace Ir(dmppy)_2_(dpp) as well as optimizing the optical effect, another doping-free tandem WOLED was constructed (device W5). As a result, device W1 could accomplish the simplified structure/short fabrication time/reduced cost/high efficiency (81.2 cd A^−1^)/low efficiency roll-off/low voltage/high luminance (44,886 cd m^−2^) trade-off, while device W5 could possess an acceptable CRI of 67. For the high performance, the doping-free CGL was effective to ensure the charge generation and separation. With the combination of KBH_4_ and Ag to modify the Bepp_2_/HAT-CN interface, the electron injection was improved, since metallic K was released by the thermal decomposition of KBH_4_ and the surface of Bepp_2_ films was not an absolute plane. Hence, a thin K-doping Bepp_2_ layer at the KBH_4_/Bepp_2_ interface was formed. Additionally, Ag functioned as electrodes for both units, also improving electron injection. On the other hand, phosphorescent emitters were adopted in each unit to not only harvest both singlet and triplet excitons but also ensure the white light.

## 4. Summary and Outlook

Since tandem device architectures can impressively boost the luminance, efficiency and lifetime, the excellent characteristics render that tandem WOLED have been extensively investigated. In this review, we have mainly focused on recent developments in tandem WOLEDs and summarized the advanced strategies to achieve high-performance tandem WOLEDs. Particularly, we have emphasized representative tandem fluorescent WOLEDs, tandem phosphorescent WOLEDs, tandem TADF WOLEDs, and tandem hybrid WOLEDs. Additionally, we have also presented doping-free tandem WOLEDs. The detailed performances for tandem WOLEDs have been described in Table 1.

Over the past few years, the performance of tandem WOLEDs has incrementally enhanced and nowadays can satisfy the demand of real commercialization for handphones, lamps and televisions. In particular, phosphorescent and TADF emitters are favorable to increase the performance. To date, there are still many challenges hindering the further development of tandem WOLEDs, including the efficiency, driving voltage, operational stability as well as viewing angle dependence. First, the PE of tandem WOLEDs still lags behind. For example, the theoretical efficiency limit for WOLEDs is about 248 lm W^−1^, indicating that there is still much room for tandem WOLEDs [225]. Besides, the voltage of tandem WOLEDs is much higher than that of single-unit WOLEDs, which increases the power consumption and easily leads to the low PE [226,227,228]. Therefore, each EL unit is required to harvest all excitons for efficient emission, while the CGL is needed to be effective for the charge generation and charge separation. In the internal physical processes, the elaborative management of the charge and exciton distribution is conducive. For the external procedures of light propagation, the utilization of outcoupling technologies can vastly enhance the efficiency as well as the lifetime [229,230,231]. This is because the use of light extraction layers can extract the light trapped by the substrate or the inner layers due to the total reflection. In addition, the light extraction layer (e.g., laminated microlens films) has been utilized to improve the CRI of tandem WOLEDs [214]. Therefore, although only a few outcoupling technologies were exclusively reported to enhance the performance of tandem WOLEDs, it is believed that the light extraction layers are promising for the further development of tandem WOLEDs. Additionally, the adoption of stable emitters and CGLs is also helpful to the lifetime.

Specifically, since the total thickness of tandem WOLEDs is very thick and the number of layers is large, the microcavity effect easily occurs because of the refractive index differences between adjacent layers (e.g., n = 1.7 for organic films, n = 1.9 for ITO, and n = 2.2 for MoO_3_). Thus, tandem WOLEDs should have the Lambertian emission and the color should be stable over all angles (regardless of the center or the corner), otherwise chromaticity angular dependence will become a serious issue since the appearance of items can be rely on the locations [232]. Additionally, the device architecture of tandem WOLEDs is usually complicated. The introduction of a doping-free technique or solution-processed fabrication method is expected to be useful. After solving the aforementioned problems, the prospect of mass production for tandem WOLEDs will be possible and the proposed solutions are also beneficial to the related optoelectronic field (e.g., display, lighting, laser, solar cell, photodetectors and sensors) [233,234,235,236,237,238]. 

## Figures and Tables

**Table 1:** Summarized performances for representative tandem WOLEDs.

WOLEDs ^a^	V_on_^b^ (v)	EQE_max_^c^ (%)	PE_max_^d^ (lm W^−1^)	CE_max_^e^ (cd A^−1^)	CIE ^f^	CRI ^g^
Ref. [72]	-	-	2.6	10.7	(0.36, 0.34)	-
Ref. [155]	-	34.9	22.7	77.0	(0.35, 0.44)	66
Ref. [162]	-	43.3	45.2	110.9	(0.34, 0.41)	-
Ref. [163]	5.7	54.3	63	-	(0.359, 0.498)	-
Ref. [163] ^h^	-	92.4	100	-	(0.336, 0.452)	-
Ref. [174]	-	74.3	24	-	(0.46, 0.43)	88.6
Ref. [174] ^h^	-	171	50	-	(0.49, 0.43)	89.4
Ref. [197]	4.0	11.6	15.8	27.7	(0.29, 0.35)	70.6
Ref. [206]	-	33	14	-	(0.38, 0.44)	82
Ref. [210]	-	~26	~40	-	(0.505, 0.422)	77.6
Ref. [210] ^h^	-	~78	~100	-	-	-
Ref. [216]	7.4	28.5	-	78.5	(0.33, 0.45)	82
Ref. [224]	5.1	-	81.2	42.9	(0.35, 0.47)	-

^a^ Representative tandem WOLEDs. ^b^ Turn-on voltage. ^c^ Peak EQE. ^d^ Peak PE. ^e^ Peak CE. ^f^ CIE coordinates at ~1000 cd m^−2^. ^g^ Peak CRI. ^h^ With the use of outcoupling technique.
